# Effects of occupational cobalt exposure on the heart in the production of cobalt and cobalt compounds: a 6-year follow-up

**DOI:** 10.1007/s00420-019-01488-3

**Published:** 2019-11-19

**Authors:** A. Linna, J. Uitti, P. Oksa, P. Toivio, V. Virtanen, H. Lindholm, M. Halkosaari, R. Sauni

**Affiliations:** 1 Kokkola Health Centre, Mariankatu 28, 67200 Kokkola, Finland; 2 Outpatient Clinic of Occupational Medicine, Tampere University Hospital, Uimalankatu 1, 33100 Tampere, Finland; 3 Finnish Institute of Occupational Health, Uimalankatu 1, 33100 Tampere, Finland; 4 Faculty of Medicine and Health Technology, Tampere University, Arvo Ylpönkatu 34, 33520 Tampere, Finland; 5 Heart Centre, Tampere University Hospital, Ensitie 4, 33520 Tampere, Finland; 6 Finnish Institute of Occupational Health, Topeliuksenkatu 41 b, 00290 Helsinki, Finland; 7 Central Hospital of Keski-Pohjanmaa, Mariankatu 16, 67200 Kokkola, Finland; 8 Ministry of Social Affairs and Health, Uimalankatu 1, 33100 Tampere, Finland

**Keywords:** Cardiomyopathy, Cobalt exposure, Work-related heart symptoms and disease, Echocardiography

## Abstract

**Objective:**

It has been suspected that cobalt is toxic to the heart. It can cause cardiotoxicity in heavily exposed humans and in experimental systems. The issue of interest for this study is whether cobalt also affects the myocardium at occupational exposure levels.

**Methods:**

To study the effect of occupational cobalt exposure on the heart, we conducted a follow-up of workers at a cobalt production plant. The workers’ hearts had been examined by echocardiography in 1999–2000. Altogether 93 exposed and 49 non-exposed workers examined in 1999–2000 were re-examined in 2006. Occupational history and health data were collected with a questionnaire. Blood pressure was measured, and electrocardiography (ECG), laboratory tests, Holter registration, and echocardiography were conducted for all participants. Analysis of covariance (ANCOVA) was used to analyse the data.

**Results:**

No differences were found between the exposed and unexposed groups for any of the echocardiographic parameters in 2006. There were no differences in the laboratory values, the ECG parameters, or the results of the Holter registration of the exposed and unexposed workers.

**Conclusions:**

Although the previous results in 2000 suggested an association between cumulative exposure to cobalt and echocardiographic findings, the results of this new cross-sectional study with a tissue Doppler 6 years later did not confirm the association in the present cohort. If cobalt exposure affects heart muscle functions at this exposure level, the effects are smaller than those caused by physiological changes due to ageing, medication, and traditional cardiovascular risk factors, such as elevated blood pressure.

## Introduction

The toxic potential of cobalt and related health risks have been investigated thoroughly in animal and human toxicity studies. Systemic toxic reactions may arise when Co ions enter the blood and lymphatic circulation and subsequently disseminate to different organs. In vitro experiments have demonstrated that ionized cobalt (Co2 +) is the primary toxic form for systemic toxicity (Leyssens et al. 2017). Various pathogenetic mechanisms related to possible cardiac effects have been suspected in hard-metal workers. According to some authors, the most likely effect is the inhibition of cellular respiration due to the inhibition of mitochondrial dehydrogenase (Seghizzi et al. 1994). Systemic Co toxicity manifests as a clinical syndrome with a variable presentation of neurological, cardiovascular, sensitizing, and endocrine symptoms, depending on the systemic Co levels (blood/urine).

Single cardiomyopathy cases have been reported among persons exposed occupationally to cobalt (Kennedy et al. 1981; Jarvis et al. 1992). In two small groups of hard-metal workers studied earlier, a weak but still significant inverse correlation was found between exposure time and the left ventricular ejection fraction as measured by radionuclide ventriculography (Horowitz et al. 1988).

A significantly lower left ventricular ejection fraction during both rest and exercise was found among men exposed to cobalt and diagnosed with hard-metal disease compared to those who had not been diagnosed with this disease (d’Adda et al. 1994) when measured by radionuclide ventriculography and echocardiography. The researchers assumed that this finding may have been due to increased diastolic pressure caused by increased wall stiffness and fibrosis due to cobalt deposits in the myocardium. In addition, hypertension and reversible electrocardiographic changes (depressed ST segment and T waves) and arrhythmias have been found more frequently among persons who have been exposed to an average cobalt concentration of 0.01 mg Co/m^3^ in hard-metal work than among persons in a reference group (Alexandersson and Atterhög 1980, 1983).

In our previous cross-sectional study in a cobalt production plant in 1999–2000, there seemed to be an association between cumulative cobalt exposure and two echocardiographic parameters, the deceleration time of the velocity (DT) of the early rapid filling wave and the left ventricular isovolumic relaxation time (IVRT). The results suggested that there were changes in the function of the myocardium during diastole (Linna et al. 2004). This finding supported the hypothesis that cobalt accumulation in the myocardium could affect myocardial function.

The aim of this 6-year follow-up was to assess the effects of cobalt exposure on functional or structural changes in the heart muscles of cobalt-exposed cobalt production workers who had been examined by echocardiography in 1999–2000.

## Methods

### Cobalt process and exposure

The cobalt plant featured in this study is located in Kokkola on the west coast of Finland. Between 1966 and 1987, cobalt powder was produced from pyrite ore concentrate. Thereafter, cobalt powder, inorganic cobalt, and nickel compounds have been produced using by-products of the metallurgic industry as raw material (Linna et al. 2004).

**Fig. 1:**
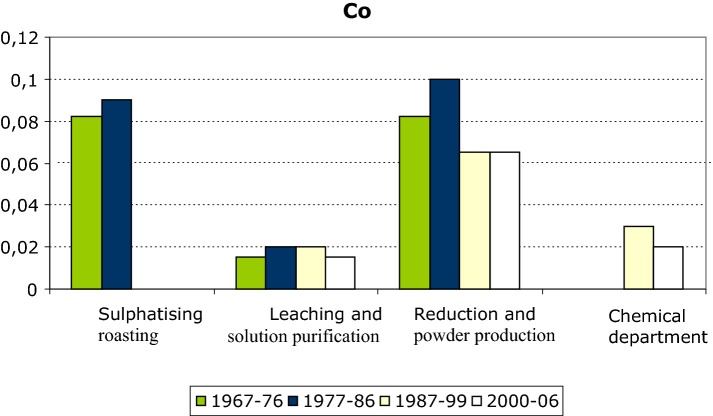
Exposure levels (mean) by departments (mg/m^3^)

Exposure to most dusts and gases in the process has been regularly monitored several times annually since 1966. Air samples have been collected by an authorized hygienist both at stationary points and with personal samplers in the workers’ breathing zones. Exposure to cobalt has varied greatly in range according to the job role even within the same department. The range has been 0.02–1.0 mgCo/m^3^ in the sulphatising roasting department, 0.01–0.05 mgCo/m^3^ in the leaching and solution purification department, 0.05–0.25 mgCo/m^3^ in the reduction and powder production department, and 0.01–0.20 mgCo/m^3^ in the chemical department (Fig. 1) (Linna et al. 2004).

The mean exposure levels to cobalt and its compounds were slightly above the current Finnish occupational exposure limit (0.05 mg/m^3^) before 1987, and they have been slightly under the limit since the new process was initiated (Linna et al. 2004). According to the biological monitoring surveillance, exposure to cobalt has been the highest in the reduction department. The company’s occupational health care unit has measured cobalt urinary concentrations (U-Co); in 1999–2000, levels of U-Co were 11–4107 nmol/l (median 240 nmol/l, *N* = 29), while in 2005–2006, they were 6–6278 nmol/l (median 230 nmol/L, *N* = 113).

Cumulative exposure to cobalt, presented as milligram-years (mg-years), was calculated for each worker using a job-exposure matrix based on ambient air measurements (Linna et al. 2004). In this study, we completed the information on cumulative exposure using the exposure data collected during the follow-up period. Between 2000 and 2006, there were no major changes in the technology or processes at the factory, and the exposure levels remained stable. In the control group (zinc plant workers), all the workers were exposed to zinc, but not to cobalt. For four-fifth of the zinc workers, the zinc exposure levels were 0.1–0.2 mg/m^3^, and for the remaining fifth, the levels were around 1 mg/m^3^.

### Design and participants

The employees who participated in this study had already been working in the cobalt plant at the end of 1999, and they had been exposed to cobalt for at least 1 year (the exposed group). The reference group had not been exposed to cobalt. In both groups, workers exposed to confounding exposure agents, such as lead and arsenic, were excluded. Persons with congenital or acquired cardiac valvular disease and those with a history of myocardial infarction were excluded from the echocardiographic analysis. In this follow-up study, we invited all the workers who had been examined by echocardiography in 1999–2000 and whose results were included in the analyses. Four employees from the earlier exposed group and one from the unexposed group had died; the cause of death of one of the exposed workers was heart infarction. The deaths of the other deceased workers were not related to cardiovascular diseases. Altogether, 19 persons were unwilling to participate or could not be contacted. Of the previously examined workers exposed to cobalt (*n* = 109), 93 (85%) were re-examined, as were 49 (86%) of the 57 who had been in the unexposed reference group. The project was separate from routine health examinations by the occupational health services. The workers were examined in 2006 within a period of 6 months. Participation was voluntary, and all participants gave their written informed consent. The study plan was approved by the Coordinating Ethics Committee of the Helsinki University Hospital District.

### Questionnaire

Data on working history at the plant after 2000 were requested with a self-administered questionnaire. The reasons for changing working tasks, jobs, or workplaces were also requested. We inquired about the level of stress, shift work, physical exercise, smoking, alcohol consumption, symptoms of chest pain or shortness of breath, diseases diagnosed by a physician (cardiovascular and pulmonary diseases, diabetes), and medication.

### Blood pressure measurement and electrocardiography

Blood pressure was measured after 10 min of rest in a sitting position using an automatic Omron 705CP (Omron Matsusaka Co Ltd, Japan), which was tested and validated by the importer before and after the study. After two measurements, the lower systolic and diastolic pressure and pulse were noted. A standard 12-lead electrocardiogram was obtained using 12-channel ECG equipment (Marquette Electronics Inc., Milwaukee WI, USA) at a paper speed of 50 mm/s. The recordings were analysed independently by two experienced clinicians, who both coded the recordings without knowing their origin (exposed/referent). Coding was performed according to the Minnesota 1982 method (Rose et al. 1982).

### Laboratory tests

As a random sample, we selected 76 persons from the exposed and unexposed groups. Serum gamma-glutamyl transferase (S-GT) and carbohydrate-deficient transferrin (S-CDT) levels were analysed. In addition, the serum lipid and glucose values of all participants, which had been recorded in health examinations during the previous 2 years, were also extracted from the files of the factories’ occupational health centre.

### Holter registration

Long-term ECG registration was recorded with Holter technics (24-h signal sampling with a three-channel device, Braemar Inc., USA). The results were interpreted by a physician (HL) who is specialized in clinical physiology. Registrations were started at the beginning of the work shift and continued overnight until the next day. The participants marked their activities and symptoms during the registration period in a diary.

### Echocardiography

Echocardiography was performed in the standard fashion using a GE Medical Systems Vivid 7/2006 ultrasound device with a 2.5-Mhz transducer and an EchoPac workstation. Conventional echocardiographic images were obtained according to the guidelines of the American Society of Echocardiography and the European Association of Cardiovascular Imaging (Lang et al. 2005). Echo exams were performed by a single operator (MH). Left ventricular mass (LVMASS) was calculated according to the formula of Devereux et al. (1986). We performed spectral Doppler measurements of blood flow by aligning the interrogating beam with the direction of flow according to anatomical and colour Doppler information. Mitral flow was obtained with the pulsed Doppler sampler (sample volume 4–5 mm in length) at the valve tips. Early diastolic filling velocity (*E*), peak atrial filling velocity (*A*), *E*/*A* ratio, E-wave deceleration time (DT), and slope were measured from the left ventricular filling recordings. We measured the isovolumic relaxation time (IVRT) using the technically better pulsed or continuous wave recording of the mitral inflow and the left ventricular outflow. No angle correction was used. Valvular regurgitation was quantified according to the colour Doppler information from multiple views. We recorded ventricular long-axis motion from the lateral and septal myocardium using pulsed-wave tissue Doppler imaging (TDI) in an apical four-chamber view with a small sample volume (2–3 mm in length) positioned in the myocardium of the basal ventricular wall, about 1 cm from the mitral annulus. Early diastolic myocardial tissue velocity (*E*_m_), diastolic myocardial tissue velocity after atrial contraction (*A*_m_), and their ratio (*E*_m_/*A*_m_) were recorded. *E*_m_ deceleration time and slope were also measured. The *E*/*E*_m_ ratio was calculated (Otto 2004). The mean of at least three consecutive beats was used. Images were stored on videotape on a Sony videocassette recorder (SVO-9500MDP), on optic disks in an Echopac Workstation, and on paper from a thermal printer (UP-890CE). The echocardiograms were analysed by a single experienced clinician without knowledge of the status of the examined persons (exposed/referent). MH performed the measurements, and another cardiologist (VV) checked the results of the measurements. The interobserver error was investigated from a sample of 30 echocardiograms, and no significant differences were observed, the coefficient of the variation of echo parameters being 2.3–18.5.

### Statistical methods

The normality of the variables was checked, and logarithmic transformation was applied if the distribution of the variable was skewed. The crude means and standard deviations are reported in the tables. The paired *t* test, the independent samples *t* test, and the McNemar test were used in the analysis of the differences between the study groups.

An analysis of covariance (ANCOVA) was used to study the echocardiograph data. A backward stepwise regression analysis was performed on all the echocardiographic parameters so that age, exposure, and their interaction were always included in the model. Body mass index was included if the outcome variable had not been divided by body surface area, and heart rate was used if the outcome variable was time related. ANCOVA was also used to study the differences in the echocardiographic parameters between the various designated exposure groups.

In the analyses, high and low exposure was determined based on being above or below the median mg-years of cobalt exposure (0.47 mg-years in 2000 and 0.55 mg-years in 2006). Age was included as a continuous variable in the ANCOVA analyses and categorized above and below 50 years and 56 years in the study in 2000 and in 2006, respectively. Independent factors, such as smoking (non-smoking vs current and ex-smoking), hypertension (as normal vs elevated (> 140/90) or as diagnosed hypertension), and competing athlete status (yes or no), were included in the model as dichotomous variables. In some calculations (see above), the use of alcohol (weekly consumption over 20 doses of 12 g of alcohol or SGT > 80), body mass index, and heart rate were included in the model as covariates because they were considered confounders. The echo values of the cross-sectional study in 2006 with the new tissue Doppler device were analysed and adjusted for the 2006 confounders. Those covariates with significant associations with outcome variables remained in the model because they were considered confounders.

The 95% confidence interval (95% CI) was calculated for the differences between two percentages. The level of significance in the ANCOVA was set equal to 0.05, but exact *p* values are reported. Computations were carried out using SPSS/Win (version 15.0) software.

## Results

The characteristics of the examined groups and calculated mg-year values are given in Table 1.

**Table 1:** Characteristics of the exposed and unexposed groups in 2000 and 2006

		Exposed group (*n* = 93)		Unexposed group (*n* = 49)	
		2000	2006	2000	2006
Age (year)	Mean (SD)	47 (8.3)	53 (8.3)	48 (7.8)	54 (7.8)
	Median (range)	50 (28–63)	56 (34–69)	50 (29–61)	56 (35–67)
Body mass index	Mean (SD)	26.9 (3.4)	27.7 (3.7)	26.9 (3.2)	27.5 (3.5)
	Median (range)	26.5 (21.6–35.0)	27.2 (20.5–37.9)	26.5 (21.6–35.0)	27.1 (21.2–36.9)
Work history (years)	Mean (SD)	22.5 (9.9)	28.1 (9.6)	23.8 (8.0)	29.5 (8.0)
	Median (range)	25 (2–34)	31 (8–40)	26 (3–25)	32 (7–41)
Exposure time to cobalt (years)^a^	Mean (SD)	20.7 (10.0)	25.7 (9.8)	–	–
	Median (range)	23.0 (0–34)	28.0 (0–39)	–	–
Exposure to cobalt (mg-years)	Mean (SD)	0.56 (0.51)	0.82 (0.79)	–	–
	Median (range)	0.45 (0.06–2.5)	0.55 (0.10–4.1)	–	–
Smoking status (%)	Non-smokers	29%	33%	33%	37%
	Ex-smokers or smokers	71%	67%	67%	63%
Consumption of alcohol (drinks/week)	Mean (SD)	5.0 (4.8)	5.9 (5.6)	6.5 (5.4)	7.1 (6.3)
	Median (range)	3.0 (0–20)	4.3 (0–26)	6.0 (0–20)	5.0 (0–25)
Competing athlete status (%)	No	91%	85%	79%	88%
	Now or earlier	9%	15%	21%	12%
Leisure-time sport activities (%)	No, never	5%	3%	4%	8%
	Yes, two times a week or less	51%	52%	46%	47%
	Yes, at least three times a week	44%	45%	50%	45%

^a^Regularly > 0.01 mg Co/m^3^

There were no significant differences in characteristics between the exposed and unexposed groups in 2006. The BMI had increased significantly in the exposed group during the follow-up. Both groups seemed to have decreased their current smoking level. Alcohol consumption had increased in both groups—significantly in the exposed group. The participants in the exposed group tended to have increased their leisure-time sport activities, but not statistically significantly (*p* = 0.065). On the contrary, the participants in the unexposed group had decreased their leisure-time sport activities, especially the duration and level of strain (data not shown). There was no significant difference in reported stress between the exposed and unexposed groups (data not shown). In the study group, 71% worked shifts, whereas 50% of the controls worked shifts.

### Symptoms, diagnosed diseases, and medication

There were no differences between the two study groups in relation to symptoms, diagnosed diseases, and medication in 2006, except for the participants in the exposed group more often reporting asthma and other pulmonary diseases (Table 2).

**Table 2:** Prevalence of reported diseases diagnosed by a physician in the exposed and unexposed groups in 2000 and 2006

	Exposed group (*n* = 93)		Unexposed group(*n* = 49)	
	2000 %	2006 %	2000 %	2006 %
Myocardial infarction	0	1	0	2
Coronary heart disease	2	3	4	4
Heart failure	0	0	0	0
Dilated heart	0	3	2	4
Heart arrhythmias	6	14**	21	16
Cardiomyopathy	0	0	0	0
Any other cardiac disease	1	3	0	0
Hypertension	23	32*	21	37**
Stroke	0	2	0	0
Claudicatio intermittens	1	3	2	2
Bronchial asthma	1	7	0	0
Chronic bronchitis	2	2	2	0
Emphysema	0	0	0	0
Other chronic lung disease	4	7	2	2

McNemar test: *p < 0.05; **p < 0.01

For both the exposed group and the unexposed group, hypertension was reported more often than 6 years earlier. In both groups, the increase in physician-diagnosed hypertension was significant during the follow-up period. In the unexposed group, the prevalence of high blood pressure had almost doubled. The proportion of persons using antihypertensive or other heart medication had increased in both groups. The use of beta-blockers was more common in the unexposed group.

Subjective complaints of irregular heartbeats (arrhythmia) had increased in the exposed group, but the prevalence was at the same level in both groups in 2006 (Table 2).

### Blood pressure, electrocardiographic findings, and laboratory tests

There were no differences in measured blood pressure, electrocardiographic findings, or laboratory tests between these two study groups in 2006, except that the exposed group had higher CDT values (Table 3). The S-CDT mean was increased in the exposed group, and the difference in the changes between the groups was significant. The S-LDL mean had decreased significantly in the unexposed group, but, again, the differences between the changes in the two groups between 2000 and 2006 were not significant (Table 3).

**Table 3:** Blood pressure, heart rate, and laboratory test results in the exposed and unexposed groups in 2000 and 2006

		Exposed group (*n* = 93)			Unexposed group (*n* = 49)			*t* test between the group differences
		2000	2006	Difference	2000	2006	Difference	*p* value
Systolic blood pressure, mmHg	Mean	135.2	142***	6.8	138.3	140.8	2.5	0.159
	SD	15.5	15.0	16.4	14.9	17.1	17.5	
Diastolic blood pressure, mmHg	Mean	87.7	90.1*	2.3	89.2	89.3	0.02	0.217
	SD	9.7	8.4	9.9	10.4	9.5	11.2	
Serum gamma-glutamyl transferase, S-GT (U/l)	Mean	50.4	52.8	2.40	35.5	41.7	6.2	0.482
	SD	56.4	47.6	29.9	15.5	31.7	27.7	
Serum carbohydrate-deficient transferrin, S-CDT (U/l)	Mean	1.5	1.6	0.09	1.6	1.3	− 0.30	0.048
	SD	0.3	0.5	0.4	0.49	0.79	0.9	
Serum glucose (mmol/l)	Mean	5.4	5.5	0.09	5.7	5.5	− 0.17	0.251
	SD	0.7	0.5	0.93	1.1	0.7	1.4	
Serum total cholesterol (mmol/l)	Mean	5.5	5.4	− 0.08	5.7	5.4	− 0.33	0.383
	SD	1.2	1.1	1.7	1.0	0.8	1.3	
Serum HDL cholesterol (mmol/l)	Mean	1.3	1.4	0.10	1.3	1.3	0.00	0.353
	SD	0.3	0.4	0.6	0.4	0.4	0.5	
Serum triglycerides (mmol/l)	Mean	1.6	1.6	− 0.01	1.6	1.8	0.11	0.661
	SD	0.9	0.8	1.2	1.1	1.7	2.1	
Serum LDL cholesterol (mmol/l)	Mean	3.6	3.4	− 0.24	3.6	3.3*	-0.38	0.578
	SD	1.0	0.9	1.4	0.9	0.7	1.1	

Paired *t* test: *p < 0.05; **p < 0.01; ***p < 0.001

Both systolic and diastolic blood pressure had risen in the exposed group in 2006 when the levels were compared to the results from 2000 (*p* < 0.05 and *p* < 0.001, respectively), but the differences in the changes in 2000–2006 between the two groups were not significant (Table 3).

There were no significant differences in the changes in the ECG findings in 2000–2006 between the two groups (Table 4).

**Table 4:** Electrocardiographic (ECG) conduction parameters for the exposed and unexposed groups in 2000 and 2006

		Exposed (*n* = 93)			Unexposed (*n* = 49)			*t* test
		2000	2006	Difference	2000	2006	Difference	*p* value
PR time, ms #	Mean	164.4	166.4		167.9	169.8		
	SD	24.5	23.6		25.8	24.3		
QRS time, ms	Mean	97.1	96.3	− 0.8	99.4	96.7	− 2.8	0.296
	SD	10.3	11.9	10.6	8.8	13.3	10.6	
QTc time, ms	Mean	408.3	408.7	0.4	410.8	410.0	-0.8	0.762
	SD	20.3	18.5	22.1	16.8	15.7	20.1	
QT time, ms	Mean	417.7	399.3***	− 18.4	416.1	414.7	− 1.5	0.062
	SD	37.8	33.4	43.6	64.0	35.2	62.0	
Pulse/min	Mean	59.1	63.9***	4.8	58.0	60.3	2.3	0.173
	SD	8.2	10.5	10.3	9.6	10.7	9.9	

# 2006 values PQ time

Paired *t* test:*p < 0.05; **p < 0.01; ***p < 0.001

### Holter registration

Holter results were obtained only from the examinations in 2006. Abnormal results in the 24-h ECG registration appeared in 10.4% of the exposed and 9.7% of the unexposed groups. The exposed group had more ventricular extrasystolic (VES) beats than the unexposed group (mean 209 (SD 1098) vs 114 (SD 362), respectively), and the unexposed group had more atrial extrasystolic beats (AES) than the exposed group (mean 216 (SD 1296) vs 87 (SD 392), respectively). Only one case of severe arrhythmia was found, and it had already been recognized in one of the referents.

### Echocardiography

One person with a history and echocardiographic signs of myocardial infarction was excluded from echocardiographic analysis in both groups. No signs of cardiomyopathy or cardiac failure/insufficiency were found clinically at the individual level.

The echocardiographic results for 2006 are presented in Table 5. Using the ANCOVA models, we considered the difference in the echo variables between the exposed and unexposed groups in 2006. In this analysis, the values of the echo parameters were adjusted to the other variables in 2006. There were no differences in any parameter measuring heart volumes, wall thickness, or muscle mass between the exposed and unexposed groups in 2006. Only A_m_ tissue was smaller in the unexposed group. There were no differences either in the DT and IVRT values between the study groups. There were no differences in any of the echocardiographic parameters in 2000 between those who participated in 2006 and those who did not (data not shown).

**Table 5:** Echocardiographic results in 2006 adjusted for age, exposure group and variables in 2006

Variable	Group									
	Exposed to cobalt ≥ 0.55 mg-year (*n* = 47)		Exposed to cobalt < 0.55 mg-year (*n* = 42)		Unexposed (*n* = 49)		*p* value			
	Mean (SD)	Adjusted mean	Mean (SD)	Adjusted mean	Mean (SD)	Adjusted mean	Age*	Exposure group	Age*Group	Covariates
LA	20.4 (2.2)	–	20.1 (2.1)	–	20.5 (2.5)	–	0.003	0.754	0.596	–
RVD	10.8 (2.2)	–	10.5 (2.5)	–	11.2 (2.1)	–	0.074	0.320	0.847	–
RVS	10.7 (1.7)	10.7	10.7 (2.1)	10.7	11.3 (1.7)	11.3	0.070	0.305	0.913	0.046 (bp)
LVEDD (mm/m^2^)	27.4 (2.5)	27.4	27.1 (2.6)	27.1	27.5 (2.5)	27.4	0.620	0.862	0.534	0.042 (alc)
LVESD (mm/m^2^)	17.1 (2.0)	17.2	16.9 (1.9)	16.9	17.1 (2.0)	17.1	0.144	0.811	0.727	0.006 (alc)
IVSD (mm/m^2^)	5.5 (0.67)	–	5.7 (0.85)	–	5.6 (0.72)	–	0.008	0.247	0.326	–
IVSS (mm/m^2^)	7.8 (0.80)	–	7.9 (1.0)	–	7.8 (0.91)	–	0.001	0.765	0.594	–
LVPWD (mm/m^2^)	4.9 (0.49)	–	5.0 (0.54)	–	4.9 (0.53)	–	0.001	0.319	0.976	–
LVPWS (mm/m^2^)	7.7 (0.77)	–	7.6 (0.85)	–	7.7 (0.81)	–	0.401	0.734	0.753	–
LVMASS	119.1 (18.4)	118.1	121.5 (26.4)	122.3	119.1 (22.6)	118.4	0.252	0.608	0.367	0.004 (bp), 0.014 (alc)
FS^a^	37.7 (4.1)	–	37.5 (5.2)	–	37.8 (5.9)	–	0.035	0.982	0.980	–
EF^a^	66.9 (5.3)	66.8	66.7 (6.8)	66.7	66.8 (7.1)	66.9	0.034	0.991	0.989	0.035 (bmi), 0.042 (alc)
IVSD + LVPWD/LVEDD^a^	0.40 (0.15)	–	0.39 (0.07)	–	0.38 (0.04)	–	0.018	0.737	0.484	–
IVRT (ms)^b^	66.9 (10.2)	66.8	68.5 (12.2)	69.4	69.6 (11.7)	68.9	0.115	0.545	0.602	0.002 (h)
DT (ms)^b^	187.4 (26.2)	186.6	183.4 (24.4)	184.2	193.9 (26.9)	192.4	0.019	0.278	0.117	0.003 (h)
*E*/*A*/ratio^b^	1.3 (0.21)	1.3	1.5 (0.44)	1.5	1.5 (0.45)	1.5	0.104	0.115	0.860	< 0.001 (h)
*E*	0.67 (0.13)	–	0.68 (0.15)	–	0.70 (0.16)	–	0.314	0.622	0.297	–
*A*	0.55 (0.15)	0.54	0.52 (0.13)	0.52	0.51 (0.11)	0.53	0.033	0.72	0.329	< 0.001 (h), 0.055 (bp), 0.056 (bmi)
*E*/*A* ratio	1.26 (0.24)	1.3	1.36 (0.33)	1.37	1.42 (0.33)	1.39	0.001	0.237	0.856	< 0.001 (h), 0.035 (bp)
DT	182.4 (26.2)	–	184.8 (26.3)	–	182.1 (25.8)	–	0.019	0.729	0.505	–
*E*_m tissue_	0.083 (0.016)	0.084	0.081 (0.020)	0.081	0.086 (0.018)	0.085	0.003	0.498	0.244	0.028 (h)
*A*_m_ tissue	0.084 (0.014)	0.082	0.077 (0.014)	0.076	0.082 (0.014)	0.083	0.007	0.028	0.466	0.023 (bp), 0.021 (bmi), < 0.001 (h)
_tissue_*E*_m/_*A*_m ratio_	1.03 (0.27)	1.06	1.12 (0.34)	1.12	1.07 (0.30)	1.04	< 0.001	0.325	0.818	0.017 (bp), < 0.001 (h)
*E*_m_DT	93.1 (14.9)	–	90.7 (14.9)	–	94.2 (19.3)	–	0.104	0.635	0.749	–
*E*/*E*_m_	8.5 (2.5)	8.4	8.6 (2.2)	8.6	8.6 (2.1)	8.6	0.021	0.83	0.314	0.002 (bmi)

Covariates in the model: exposure, age and their interaction are permanently in the model, in addition, following adjusting covariates have been put on the model, only the significant associations are shown: smoking, blood pressure > 140/90 in 2006 or earlier hypertension diagnosed by physician (bp), athlete sports (as)

*E* early filling wave velocity, *DT* deceleration time, *A* atrial filling wave velocity, *E/A ratio* ratio between velocity of early and atrial filling, *E/E*_*m*_ early filling wave velocity/tissue Doppler *E* velocity, *E*_*m*_*DT* deceleration time, *E*_*m*_ tissue early filling wave velocity, *A*_*m*_ tissue atrial filling wave velocity, *E*_m_/*A*_m_ ratio, *LA* left atrium, *RVD* right ventricle (diastolic), *RVS* right ventricle (systolic), *LVEDD* left ventricular end diastolic diameter, *LVESD* left ventricular end systolic diameter, *IVSD* interventricular septum (diastolic), *IVSS* interventricular septum (systolic), *LVPWD* left ventricular posterior wall (diastolic), *LVPWS* left ventricular posterior wall (systolic), *LVMASS* left ventricular mass, *FS* fractional shortening, *EF* ejection fraction, *AO* aorta, *LVOT* left ventricular outflow tract, *IVRT* isovolumic relaxation time

^a^Body mass index (bmi) (adjusted in the model in addition, only the significant associations are shown), ^b^bmi, heart rate in echo (h) (adjusted in the model in addition, only the significant associations are shown) age groups <56v ja>=56v in 2006

## Discussion

This study is the first follow-up cohort study to assess the effects of occupational cobalt exposure on the cardiovascular system. We carried out a 6-year follow-up with echocardiography in a cobalt production plant. Cobalt exposure was not associated with detrimental effects on the function or structure of the heart muscle, and it did not seem to have caused damage to the cardiovascular system.

We performed a cross-sectional study in 2000 (Linna et al. 2004) and repeated it in 2006 for those who had been examined with echocardiography in 2000. There are several strengths to our study. All the participants were re-examined by the same investigator (a cardiologist). Our study was based on a large, occupationally exposed study population, and we could take the advantage of a reliable modern method, namely echocardiography with a tissue Doppler. Participation rate is high (85% and 86% in the exposed and unexposed groups, respectively). Another strength of the study was the exposure assessment, which was based on regular industrial hygienic measurement over decades. The weaknesses of the study include the limited number of participants in the unexposed group, which diminished the power of the study, and the subjective nature of the health data from the questionnaire. On the other hand, we decided to use the results of the laboratory tests as background variables to assess the possible changes in the lifestyle profiles of the groups in the follow-up. These test results were in concordance with the questionnaire results.

We have no accurate personal health information on those who did not participate in the follow-up. According to the information from the occupational health centre, no clinical cases of cardiomyopathy were found in this factory during the follow-up period. Occupational asthma has been associated with cobalt exposure, and we have published a case series on cobalt-induced asthma previously (Sauni et al. 2010). However, there is no evidence that occupational asthma is associated with a risk of heart failure. We could not see differences in any of the echocardiographic parameters in 2000 between those who participated in 2006 and those who did not. Thus, there does not seem to be a remarkable selection bias.

Echocardiography with a tissue Doppler was used in 2006, because we wanted to confirm previous findings with reliable, modern, and high-quality technology. Therefore, the results of the echo method with the tissue Doppler were not comparable with the previous Doppler echocardiography. The results in 2006 have, therefore, been analysed as a new cross-sectional study design using similar statistical models as employed in 2000.

Exposure levels of cobalt and other impurities in the air have been monitored regularly in the cobalt factory featured in our study, and this enabled individual assessment of the cumulative exposure to cobalt (Linna et al. 2004). The working environment in this study design offered no exposure to agents other than cobalt that would have had harmful effects on the cardiovascular system. Exposure to zinc does not cause effects on the heart muscle, and the group exposed to zinc was an epidemiologically ideal reference group, because it came from a factory in the same industrial area with the same recruitment criteria and similar pre-employment examination by the same occupational health unit.

These results should be applied and generalized to the exposure levels monitored in this factory. The mean exposure levels of cobalt and its compounds in the plant were slightly over the current Finnish occupational exposure limit (0.05 mg/m^3^) before 1987, but they have been slightly under the limit since the new process was initiated. During the early years of cobalt production, the cobalt levels may have been considerably higher (i.e. over 1 mg/m^3^), especially in the roasting department. Finnish occupational limits have often been exceeded also in the reduction and powder production departments. In the 2000s, the concentrations of cobalt in ambient air tended to decrease. The follow-up in biomonitoring U-Co showed that exposure remained at the same level.

A search of the literature found one Belgian epidemiologic cross-sectional study that did not find an association between cobalt exposure and incipient signs of cardiomyopathy (Lantin et al. 2013). The researchers studied 256 male refinery workers with electro- and echocardiography. Their results were adjusted to an exposure index reflecting long-term exposure to cobalt. No dose–response relationship was found between exposure to cobalt and parameters reflecting possible dilated cardiomyopathy in their exposed study population.

In our cross-sectional study in 2000, we found that higher cobalt exposure was associated with altered left ventricular diastolic function as measured by echocardiography (Linna et al. 2004) The isovolumic relaxation time (IVRT) was prolonged in the highly exposed group when compared with that of the less exposed and reference groups, and a prolonged deceleration time (DT) was observed among the exposed persons. These findings were in concordance with those of D’Adda et al. (1994), who reported that an accumulation of cobalt in the myocardium might result in increased myocardial stiffness. Contrary to the previous echo findings, in 2006, we found that DT and IVRT were similar in the exposed and unexposed groups. In the 2006 results, the use of the tissue Doppler, when adjusted for confounding variables, did not show any differences between the exposed and unexposed groups. Only the A_m_ tissue was smaller in the unexposed group; however, the higher age and heart rate (time-related variables) best explained these values. Furthermore, no significant differences were found between the exposed and unexposed workers in the laboratory values, ECG parameters—including heart rate—or Holter registration. These findings support the no-effect results of cobalt exposure found by the echocardiography.

The differences in the echo parameters between the study groups in 2000 were minimal when compared with the pathological findings in the context of clinical practice. Small changes in echo parameters are apt to become significant in group-level comparisons in epidemiological studies.

When there was no progression of the earlier changes in the exposed group compared to unexposed group, we consider the earlier findings from 2000 in the exposed group to have developed randomly; while these findings were statistically significant, they were not clinically significant. The changes in the echocardiographic parameters may have been associated with the process of ageing and its physiological changes on the cardiovascular system, which may explain many of our findings in these two cross-sectional studies with a 6-year interval.

In the questionnaire data, background factors—such as lifestyle habits—had changed in both groups during the follow-up. The BMI had increased in both groups, and the use of alcohol was more common among the unexposed participants. The exposed participants had increased their leisure-time sport activities, while unexposed participants had decreased such activities. It seemed that the lifestyle habits of the unexposed group tended to have changed more in an unhealthy direction compared to the exposed group. In this middle-aged male population, possibly the largest effect on heart function was due to hypertension and its medication. This explanation is supported by the more frequent reports of physician-diagnosed hypertension in both groups, which was present even more frequently in the unexposed group. The unexposed group used more beta-blockers as antihypertensive medication compared to the exposed group (18% vs 11%). Many of the echo variables are dependent on hypertension and heart rate and, therefore, we adjusted the variables to both factors in the analysis.

It is possible that our previous findings of a possible increased stiffness of the myocardium were not associated with cobalt exposure. It seems likely that even if cobalt exposure has a small effect on heart function, it is unlikely that this effect can be demonstrated in a middle-aged male population with many changing risk factors for cardiovascular diseases and medication affecting heart function. The exposed and unexposed groups did not differ from each other clinically or statistically significantly with respect to any echo variable, meaning we could not conclude there were detrimental effects on the heart due to exposure to cobalt. Based on the findings of this study, there is no need to add heart-specific tests to the health surveillance scheme of the cobalt-exposed workers when exposure levels do not exceed those reported in this study.

## Conclusions

We found no cardiac dysfunction that could be attributed to cobalt exposure. If there are effects of cobalt exposure on the functions of the heart muscle, they are probably smaller than the effects of ageing, medication, and traditional cardiovascular risk factors, such as elevated blood pressure.
